# Spatiotemporal orchestration of the salivary gland immune microenvironment in Sjögren’s disease: a multidimensional framework of epithelial licensing, lymphoid neogenesis, and stromal remodeling

**DOI:** 10.3389/fimmu.2026.1863280

**Published:** 2026-07-15

**Authors:** Shouze Ren, Qiwei Li, Rui Niu, Xixi Liu, Peng Zhou, Xiadong Yang, Hua Liang

**Affiliations:** 1 Heilongjiang University of Chinese Medicine, Harbin, China; 2 Beijing Mentougou District Hospital of Traditional Chinese Medicine, Beijing, China

**Keywords:** epithelial licensing, immune microenvironment, precision medicine, Sjögren’s disease, stromal remodeling, structural locking, tertiary lymphoid structure

## Abstract

Sjögren’s disease (SjD) is a chronic autoimmune disease characterized by lymphocytic infiltration of the exocrine glands and progressive secretory dysfunction. Although the major molecular abnormalities in SjD, including prominent type I interferon activation and abnormal B-cell responses, are now better defined, these findings have not consistently translated into effective disease-modifying therapy. A persistent gap therefore remains between biological target engagement and clinical benefit. This review argues that this gap may partly reflect a mismatch between current pathological models of SjD and the way clinical trials are designed. Conventional frameworks often treat SjD as a relatively homogeneous inflammatory disease and rely heavily on systemic disease-activity measures. Such approaches may underrepresent the spatiotemporal evolution of the salivary gland microenvironment and the heterogeneity of treatment responses across tissue states. Drawing on recent evidence, including single-cell RNA sequencing, spatial transcriptomics, and longitudinal histological studies, we propose a tissue-centered interpretive framework for SjD. This framework highlights three partially overlapping dimensions: epithelial activation states linked to nucleic-acid-sensing and interferon-related programs; lymphoid organization and B-cell-supportive niches associated with IFN-BAFF and Tfh/Tph-related activity; and later structural remodeling associated with fibrosis, impaired regeneration, and reduced functional reversibility. These dimensions are used here as interpretive constructs rather than as validated clinical staging categories. On this basis, precision medicine in SjD may need to move beyond a uniform anti-inflammatory strategy toward mechanism-based stratification anchored in tissue state and disease stage. Biomarkers such as salivary gland ultrasonography, TLS-related features, and microenvironment-linked molecular readouts may help refine patient stratification, endpoint selection, and clinical trial design, although these applications still require prospective validation.

## 1 Introduction

### 1.1 Epidemiological burden and systemic heterogeneity

SjD is a complex systemic autoimmune disease that primarily affects the exocrine glands and commonly causes xerostomia and keratoconjunctivitis sicca (1). Depending on the classification criteria used, its reported prevalence ranges from approximately 0.01% to 0.72% worldwide (2). SjD shows a marked female predominance, especially among postmenopausal women, with a female-to-male ratio of about 9:1 (3). Although dryness is the most familiar clinical presentation, the burden of disease extends well beyond oral and ocular symptoms (4). About 30% to 40% of patients develop extra-glandular manifestations, including arthralgia, pulmonary involvement, neurologic disease, and renal injury, which further increase morbidity and treatment complexity (5).

SjD is also associated with an increased risk of lymphoma (particularly B-cell non-Hodgkin’s lymphoma [NHL]), with the risk assessment value being much higher than that of the general population (6). Current evidence indicates that chronic B-cell activation, progressive changes in lymphoid tissue structure, and high-risk clinicopathologic features may promote the occurrence of lymphoma in some patients (7–10). Therefore, the association between SjD and lymphoma has important implications for risk stratification, follow-up management, and the selection of treatment timing.

In terms of economic burden, the long-term treatment and management of SjD require a significant amount of resources. Besides symptomatic treatments such as artificial tears and oral lubricants, patients may also need to use immunosuppressants for a long time to control systemic symptoms (11). The chronic course of this disease and its impact on multiple organs make it a highly burdensome disease state, which not only increases medical costs but also has a profound impact on the social function of patients (12).

### 1.2 Genetic susceptibility and the evolution of pathogenesis

Over the past two decades, research in genetics and immunology has significantly deepened our understanding of the pathogenesis of SjD. Genome-wide association studies (GWAS) have confirmed that this disease has a polygenic pathogenic background, with the HLA-DRB1 and HLA-DQB1 loci in the MHC II region being the most definitive related gene loci; these variations are not only associated with disease susceptibility but also affect the regulation of peripheral immune responses (13, 14). Beyond the MHC region, gene loci such as IRF5, STAT4, and OAS1 related to innate immune perception and interferon biology may also further influence the tendency of the body to have an excessive immune response to endogenous or exogenous stimuli (15).

At the molecular level, one of the most reproducible findings in SjD is the interferon (IFN) signature. In many patients, sustained upregulation of type I interferon-stimulated genes (ISGs) can be detected in peripheral blood and salivary gland tissue. This pattern is consistent with chronic antiviral-like immune activation even in the absence of proven ongoing viral infection, and it provides one important link between innate and adaptive immune dysregulation in the disease (16).

A more cautious interpretation is that viral-mimicry-like mechanisms may contribute to chronic immune activation in SjD. Specifically, endogenous nucleic acids may be misrecognized as exogenous viral material through receptors such as TLR3, TLR7, and TLR9, or through abnormal activation of cytoplasmic sensors such as RIG-I, MDA5, and cGAS, thereby triggering IFN responses. This framework is conceptually useful because it provides one possible explanation for how sterile tissue stress may be translated into persistent innate immune activation (17).

### 1.3 The clinical trial dilemma: disconnect between biological activity and clinical benefit

Although understanding of SjD molecular mechanisms has advanced considerably, translation into effective disease-modifying therapy remains difficult (18). Several trials targeting B-cell depletion, BAFF-dependent survival signals, or co-stimulatory pathways have shown biological activity, yet improvement in key clinical endpoints such as dryness and fatigue has often been limited or inconsistent (19, 20).

For instance, the JOQUER and TRACTISS trials respectively evaluated the application of hydroxychloroquine and rituximab in primary SjD, but neither of the two trials demonstrated significant improvement in core symptoms such as dryness and fatigue at the primary endpoints (20, 21). However, some studies still indicate that immunomodulatory therapy can alter peripheral immune activation or inflammation-related biomarkers without simultaneously restoring glandular secretion function (22). This situation suggests that acting only on the target may be insufficient, and factors such as treatment timing, disease course, tissue damage, endpoint assessment methods, and patient stratification may all affect the trial results (20–22).

### 1.4 The core problem: endpoint mismatch and limitations of static models

One recurring limitation in current research and clinical trial design lies in possible mismatch between selected endpoints and biologically relevant target-organ change. ESSDAI, as a systemic activity score for SjD, focuses mainly on extra-glandular manifestations and may not fully reflect glandular microenvironmental change, secretory reserve, or symptom experience (23–25). As a result, some interventions may reduce systemic inflammatory activity without producing parallel improvement in dryness, fatigue, or glandular function. However, endpoint mismatch should not be treated as the only explanation for trial failure, because patient heterogeneity, disease duration, irreversible glandular damage, placebo effects, background therapy, and target biology may all contribute.

Traditional pathological models often describe SjD as a relatively static and homogeneous inflammatory disease, thus underestimating the spatiotemporal heterogeneity within the gland (26). Single-cell RNA sequencing and spatial omics studies support the existence of a more dynamic immune microenvironment that changes over time and across tissue regions (27–29). This in itself does not deny the effectiveness of existing treatment strategies, but it does indicate that clinical efficacy may be difficult to judge accurately if glandular injury, inflammatory activity, and lesion reversibility are not assessed together (30).

To avoid overinterpretation of emerging data, the conceptual language used throughout this review requires explicit definition. Several recurrent terms should therefore be understood as interpretive constructs rather than as validated clinicopathologic categories. In this context, “epithelial licensing” refers to inflammation-responsive epithelial states associated with altered immune-interaction capacity; “pathological ratchet effect” refers to the possibility that once chronic immune-supportive circuits are established, disease persistence becomes progressively less reversible; and “structural locking” refers to later tissue states in which accumulated architectural damage constrains functional recovery even when inflammatory activity is reduced. Related descriptions of regenerative decline or functionally constrained disease are likewise intended to summarize tissue-level observations rather than define discrete validated stages. These terms are used here to organize current evidence, highlight mechanistic hypotheses, and support testable stage-aware interpretation, not to introduce a formal staging system.

## 2 The epithelial checkpoint: from passive targets to immune organizers

In the pathogenesis of SjD, salivary gland epithelial cells (SGECs) should no longer be simply regarded as passive targets of immune attack (31). Increasing evidence indicates that, especially in the early or active lesion stages, these epithelial cells may adopt inflammation-responsive states, thereby helping to shape the local immune microenvironment. In this case, SGECs not only integrate local inflammatory signals and alter the secretion levels of chemokines, but also participate in the local immune regulatory process, rather than merely passively enduring damage.

From a mechanistic perspective, this “epithelial checkpoint” can be understood as a form of functional remodeling driven by transcriptional reprogramming. After exposure to interferons, pattern-recognition signals, and local inflammatory mediators, salivary gland epithelial cells (SGECs) may abnormally express cytokines, chemokines, and antigen-presentation-related molecules, thereby altering the homing, interaction, and retention of immune cells within the tissue (32, 33). A more restrained interpretation is that the key pathological changes in SjD reflect not only immune-cell infiltration but also epithelial-state changes that may help sustain the local inflammatory microenvironment. In this sense, SGECs may be viewed as an important interface linking innate immune sensing, local inflammatory amplification, and later lymphoid organization.

### 2.1 Topological distribution and local autocrine loops of the interferon signature

The interferon signature is one of the important molecular markers of SjD, involving a group of interferon-stimulated genes (ISGs) regulated by type I interferons (IFN-α/β) and type II interferons (IFN-γ). Studies have shown that IFN-related gene upregulation can be detected in salivary gland tissue and peripheral blood of patients with SjD, but they are not completely consistent. Among them, peripheral blood is more often characterized by type I IFN, while salivary gland tissue can show a more prominent local IFN activation pattern (34). This difference between local and systemic IFN signals is consistent with compartmentalized target-organ inflammation, but does not by itself prove epithelial autonomy or causal primacy (35, 36).

The latest single-cell and spatial transcriptomics studies have further revealed that there can be significant local upregulation of ISGs in salivary gland tissues, even though such changes may not be fully reflected by peripheral blood characteristics (37). Meanwhile, experimental studies have shown that SGECs can participate in the local amplification of type I IFN through the LAMP3-TLR7-related pathway, supporting the existence of a persistent local IFN positive feedback loop within the salivary gland microenvironment (38). This phenomenon is consistent with the possibility that abnormal IFN activity in SjD is shaped partly within the target organ, although current evidence remains insufficient to establish a fully autonomous local circuit.

From a spatial perspective, histologic, flow-cytometric, and transcriptomic studies suggest that enhanced ISG expression in salivary glands is concentrated around epithelial compartments and inflammatory infiltrates rather than being uniformly distributed (39). Local IFN-related programs have also been associated with B-cell accumulation and with tissue organization favoring lymphoid aggregation (40). Accordingly, the pathological value of IFN signatures may lie not only in reflecting inflammatory intensity but also in marking tissue contexts that support immune-cell retention, interaction, and sustained activation.

**Figure 1:**
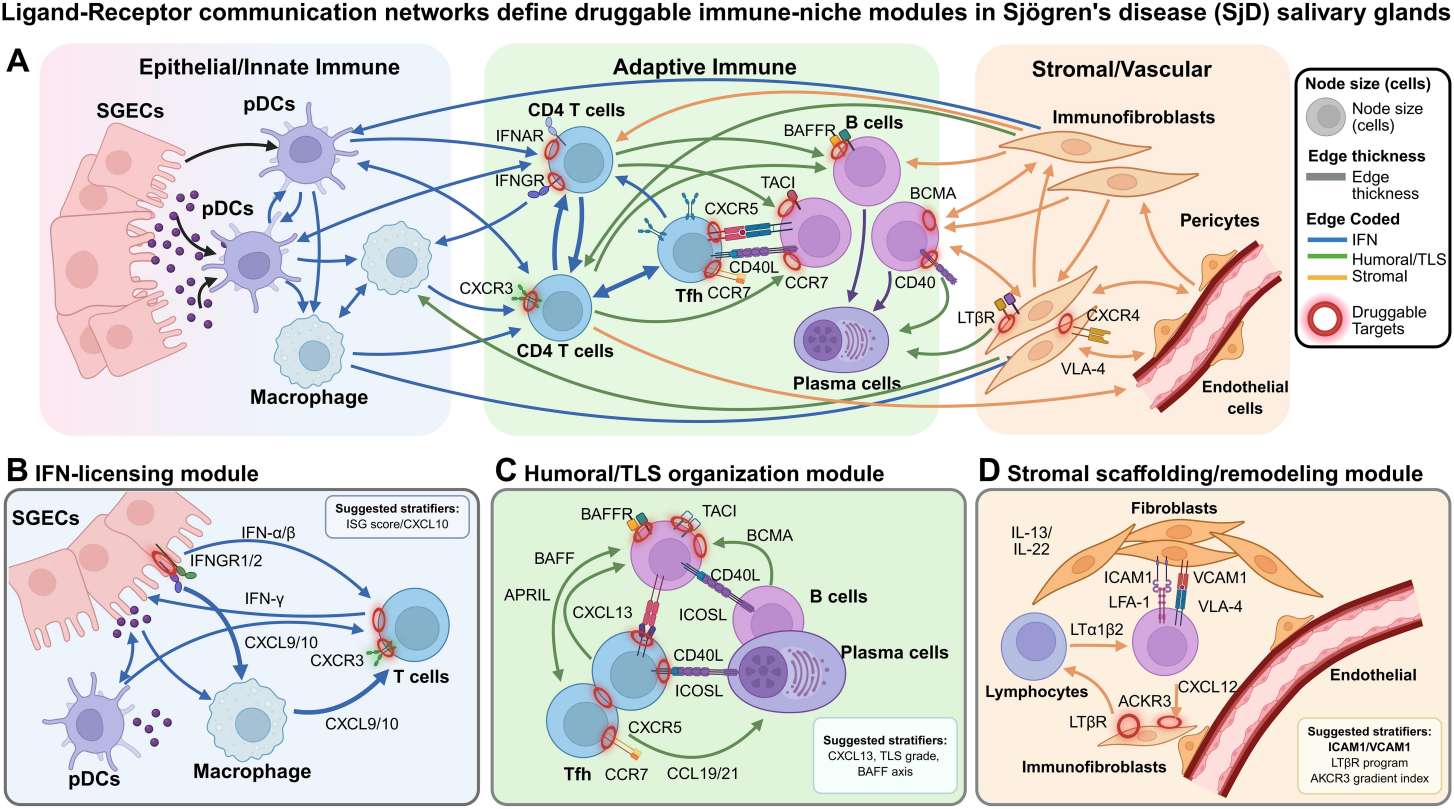
Illustrative ligand-receptor communication network within the salivary gland immune microenvironment in SjD. **(A)** Interactions among epithelial, immune, stromal, and vascular compartments, with candidate therapeutic nodes highlighted. **(B)** IFN-licensing-related pathways among SGECs, pDCs, macrophages, and T cells. **(C)** Humoral and tertiary lymphoid structure organization involving Tfh cells, B cells, and plasma cells. **(D)** Stromal scaffolding involving lymphocytes, fibroblasts, immunofibroblasts, and endothelial cells. The diagram is conceptual and does not imply that all interactions are equally validated. Created in BioRender. Ren, S. (2026) https://BioRender.com/lh0ahc8

Based on this understanding, the value of IFN signals in SjD should not be limited to monitoring at the level of peripheral blood biomarkers alone. Compared with the simple assessment of circulating IFN activation, the analysis of local IFN programs and their spatial distribution in salivary glands may be more helpful in identifying tissue-proximal abnormalities in the early stage of the disease, and provide a more pathologically-relevant basis for patient stratification and treatment response evaluation (41). In this sense, interferon-related features may be better understood as candidate indicators of tissue-state organization than as proof that epithelial cells have acquired stable immune-organizing autonomy (**Figure 1**).

### 2.2 Viral mimicry and epigenetic derepression: the molecular origins of sterile inflammation

In SjD, the persistence of local inflammation does not necessarily depend on exogenous pathogen stimulation. Accumulating evidence suggests that there may be a state of “viral mimicry” in target organs, in which the innate immune system responds to abnormally exposed or transcriptionally derepressed endogenous nucleic acids as if they were virus-associated molecular patterns, thereby continuously triggering antiviral immune responses (42, 43). The significance of this mechanism is that it provides a more explanatory molecular framework for the long-standing sterile inflammation in SjD: even in the absence of definitive exogenous infection, glandular tissue itself may maintain an interferon-driven inflammatory program through aberrant nucleic acid sensing.

The key step of this process occurs at the level of SGECs. Studies suggest that SGECs in SjD can continuously upregulate a variety of nucleic acid sensing molecules, including cytoplasmic nucleic acid sensors RIG-I, MDA5, cGAS, and endosome-related TLR3, TLR7, and TLR9 (44). These receptors are primarily used to recognize viral RNA or DNA, but under pathological conditions, they may have abnormal responses to endogenous nucleic acids, thereby activating downstream MAVS or STING-related pathways and inducing type I interferon and interferon-stimulated gene expression. Thus, abnormal interferon activity in SjD may reflect not only peripheral immune activation but also ongoing target-organ responses to aberrantly derived endogenous nucleic acids.

Epigenetic derepression is one plausible mechanism for the generation of such aberrant endogenous nucleic acids. Previous studies have suggested that abnormal DNA methylation, disordered histone modification, and broader transcriptional imbalance can lead to derepression of repetitive sequences such as human endogenous retroviruses (HERVs) and long interspersed nuclear elements (LINE-1) (45). These abnormal transcriptional products can form double-stranded RNA (dsRNA) with immunogenic properties or other abnormal nucleic acid intermediates, thereby mimicking molecular patterns seen during viral replication and activating RIG-I/MDA5-related antiviral pathways. Meanwhile, mitochondrial stress and mitochondrial DNA release may also provide additional endogenous ligands for the cGAS-STING pathway, further amplifying the local interferon cascade (46, 47). Thus, “viral mimicry” in this context does not imply persistent exogenous viral infection, but rather abnormal re-exposure of endogenous nucleic-acid information that can sustain antiviral-like inflammatory signaling within tissue.

Pathologically, this framework shifts attention away from chronic inflammation as a process sustained only by continuous peripheral immune-cell input. It raises the possibility that the target organ itself may contribute to persistence by generating endogenous immune stimuli through abnormal nucleic-acid sensing. In that sense, SGECs may not only respond to IFN signals but may also help sustain local IFN-related programs, although the extent of this contribution still needs direct functional validation.

The existing experimental studies have, to a certain extent, supported this framework. Multiple studies have indicated that the expression of HERVs, LINE-1 and nucleic acid sensing-related molecules in SjD can be abnormally elevated, and is associated with enhanced expression of IFN-stimulated genes (ISGs) and tissue inflammatory characteristics (48). Although the definition of the specific molecular source, dominant pathway and cell specificity of different studies is still not completely consistent, the overall trend supports the view that viral mimicry and epigenetic derepression are plausible upstream contributors to local immune activation in SjD, although they cannot yet be regarded as established dominant mechanisms.

This framework may also have translational implications, although these should be framed cautiously. If the local inflammation of SjD is partly caused by abnormal nucleic acid sensing and aberrant activation of antiviral-like programs, then downstream anti-inflammatory inhibition alone may not be sufficient to interrupt self-sustaining tissue inflammation. In contrast, interventions targeting upstream interferon pathways, nucleic acid sensing nodes, or related epigenetic derepression mechanisms may theoretically offer a better chance of interrupting sustained sterile inflammatory signaling (49, 50). Future research can further focus on HERVs, LINE-1, mitochondrial DNA leakage and cGAS-STING/RIG-I-MAVS related pathways to more clearly define which patients truly have a viral-mimicry-dominant tissue state. Therefore, more mechanistically targeted patient stratification and treatment design can be promoted (51–54).

### 2.3 Epithelial licensing: non-professional antigen presentation and ectopic chemokine synthesis

In SjD, SGECs should no longer be regarded merely as passive damaged target cells. Increasing evidence suggests that under sustained inflammatory stimulation and transcriptional reprogramming, SGECs may enter a state referred to here as “epithelial licensing”, shifting from primarily structural secretory and barrier cells toward inflammation-responsive cells that may participate in local immune organization. The key significance is not only the expression of several immune-related molecules, but also the further transformation of epithelial cells from “inflammatory responders” to cells that may help shape the local immune microenvironment: on the one hand, they may alter local lymphocyte distribution by abnormal expression of chemokines; on the other hand, they may help support local T-cell activation by acquiring incomplete antigen-presentation-like functions, thereby contributing to progression from diffuse inflammation toward more organized chronic immune responses (55, 56).

This process is first manifested in the abnormal activation of the chemotactic factor program. Under normal circumstances, the homing of lymphocytes and the maintenance of follicular structure mainly rely on a specific immune matrix network. However, in SjD, SGECs may also contribute to this altered chemotactic environment. It is particularly noteworthy that molecules such as CXCL13, CCL19, and CCL21, which are closely related to the recruitment of lymphocytes and the formation of tertiary lymphoid structures (TLS), show an upward trend in the diseased glandular tissues. CXCL13, as the core chemokine of CXCR5^+ B cell homing, is usually regarded as an important component of the FDC-related network. However, in the inflammatory environment of SjD, epithelial cells may be involved in the establishment of a local CXCL13-related chemotactic axis, thereby potentially providing spatial conditions for B-cell aggregation and subsequent TLS organization (57, 58). Therefore, the role of SGECs is not merely the secretion of a chemotactic factor, but rather the reshaping of local migratory cues, helping shift glandular tissue toward a microenvironment that supports the retention, aggregation, and interaction of immune cells.

In addition to their chemotactic function, SGECs can also upregulate MHC II molecules and some co-stimulatory related molecules, such as CD40, under the stimulation of inflammatory factors like IFN-γ, thereby acquiring non-professional antigen-presenting-like features (59). However, this phenomenon is better understood as a biased and incomplete immune program rather than as reproduction of full professional antigen-presenting-cell function. Compared with professional APCs such as dendritic cells, the configuration presented by SGECs is more likely to be a localized inflammatory amplification program: they possess certain antigen presentation and T cell activation support capabilities, but may not simultaneously possess the complete regulatory mechanisms required to maintain peripheral tolerance. Accordingly, this “epithelial licensing” state may be less relevant to effective antigen clearance than to low-grade but persistent local immune activation, with prolonged retention of T-cell responses within the gland (60–62). From a pathological perspective, this imbalance is particularly significant because it suggests that the local immune abnormalities in SjD are not entirely dependent on the continuous input of peripheral immune cells, but may be maintained to a certain extent by the immune amplification capacity obtained by the glands themselves.

Looking further, the epithelial permissive state may mark a potential “plasticity window” in SjD, in which structural reserve is still partly preserved but local immune circuits are becoming more self-sustaining. If so, the epithelial-lymphocyte interaction axis may be relevant not only to mechanistic study but also as a candidate target for earlier intervention. In this setting, treatment effects may be more likely to appear first as changes in local chemotactic networks, TLS organization, or tissue-proximal immune activity than as immediate recovery of salivary flow (63–66).

**Figure 2:**
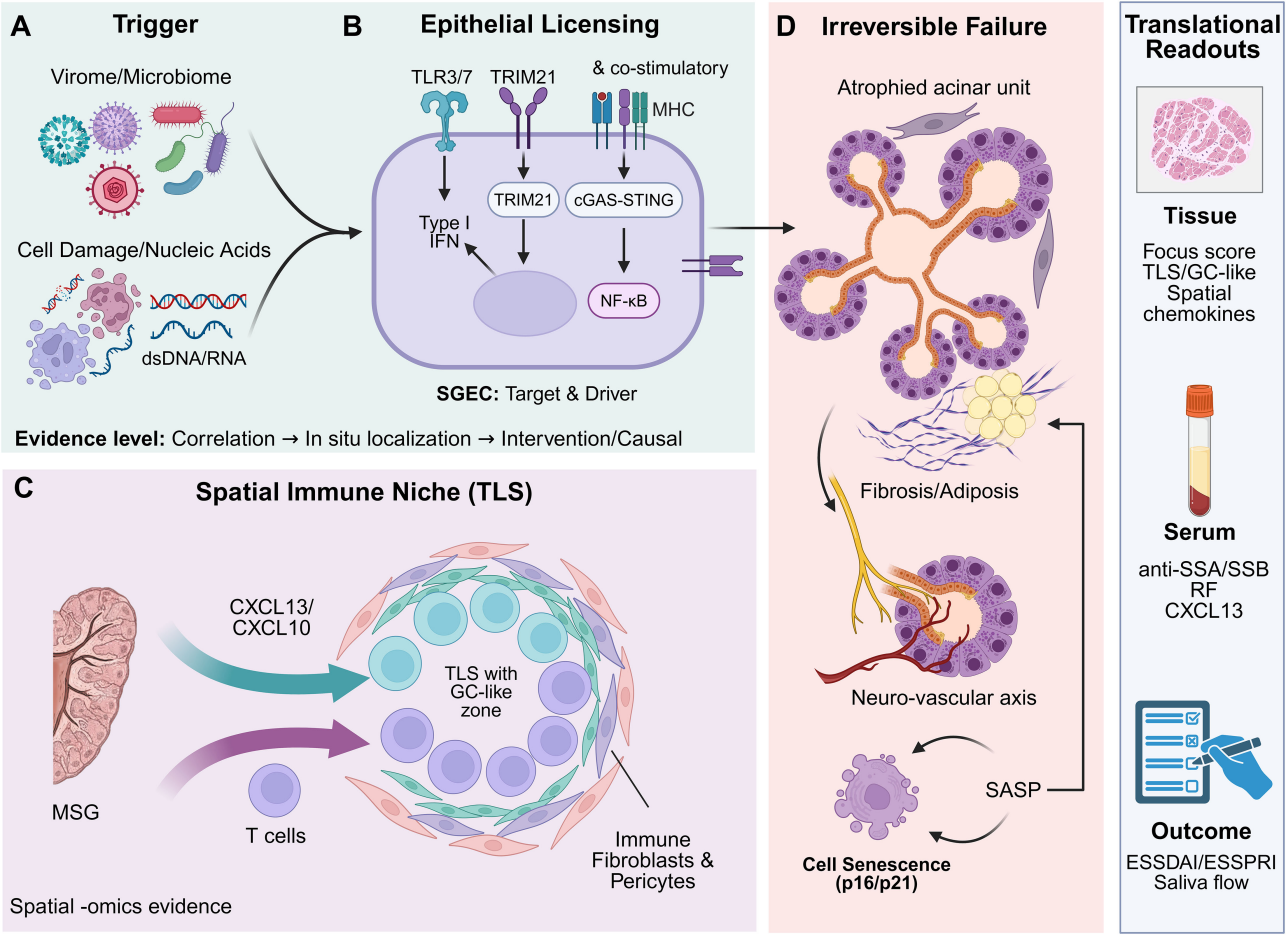
Conceptual relationship between epithelial licensing and the local immune microenvironment. **(A)** Putative triggers, including microbes and nucleic acids, that initiate immune activation. **(B)** Epithelial licensing through signaling pathways in glandular epithelial cells. **(C)** Spatial immune niche organization, including tertiary lymphoid structure-related arrangements of immune and stromal cells. **(D)** Irreversible tissue failure characterized by atrophy, fibrosis, neurovascular alterations, and cell senescence. The rightmost column summarizes illustrative translational readouts. The state transitions shown are hypothesis-generating rather than validated lineage steps. Created in BioRender. Ren, S. (2026) https://BioRender.com/z9b48of

From a translational perspective, these epithelial-state changes may help define a candidate tissue-level interpretive framework linking local immune organization to later chronicity and treatment stratification (**Figure 2**).

### 2.4 Coupling of the IFN-BAFF axis and the pathological ratchet effect

In SjD, the functional coupling between interferon (IFN) signaling and B-cell activation factor (BAFF) constitutes a crucial hub that links the activation of innate immunity with the chronicity of adaptive immunity (67). In a local inflammatory environment, SGECs and other resident cells can be activated by the IFN signal and activate the JAK-STAT pathway, thereby inducing BAFF expression. This reduces the threshold for B cell activation and survival, and promotes the retention and expansion of autoreactive B cells (68, 69). The result is not simply linear amplification of a single inflammatory signal, but a self-reinforcing circuit in which IFN promotes BAFF expression and BAFF helps sustain abnormal B-cell responses and local immune activation.

The importance of this coupling lies in the possibility that transient inflammatory inputs may evolve into a sustained B-cell-supportive environment. Studies have shown that BAFF expression is significantly increased in salivary gland tissues of SjD, especially in the areas of immune cell infiltration and lymphocyte aggregation. Its expression level is not only higher than that of normal controls, but also correlates with the degree of local inflammatory infiltration (70, 71). This suggests that BAFF may be involved not only in B-cell activation, but also in maintaining a tissue microenvironment that supports B-cell survival, differentiation, and local interaction. Some studies suggest that B-cell targeted therapies such as rituximab can affect local B-cell activation-related pathways and certain biological indicators. However, the clinical benefits, especially in terms of symptoms such as dry mouth and fatigue, are not consistent. Meanwhile, as the disease progresses, the BAFF-related activation network may gradually stabilize and integrate into the local pathological microenvironment, making it more difficult to reverse the established immune maintenance circuit by blocking a single node (72–74).

From this perspective, the so-called “pathological ratchet effect” refers to the possibility that, once the B-cell support network, local antigen presentation, and inflammatory amplification processes linked to the IFN-BAFF axis are established, disease persistence becomes progressively less dependent on the initial trigger. Once such circuits are established, later interventions may improve upstream signals without fully restoring a more reversible tissue state. Clinical observations are compatible with this interpretation: BAFF inhibition therapy such as belimumab can reduce biomarkers related to B-cell activation, but clinical benefits, especially in patient-reported symptoms, do not always parallel biological improvement. The BELISS open-label study suggested improvement in some clinical and biomarker outcomes, whereas later controlled studies showed that biomarker modulation did not consistently translate into improvement in dry mouth or other patient-reported outcomes (75, 76). Such results do not necessarily mean that the target is irrelevant; they may instead reflect a mismatch among intervention timing, tissue condition, disease duration, and pathological stage. This concept should therefore be regarded as a mechanistic interpretation of chronicity rather than as a validated biological stage.

The significance of the IFN-BAFF axis is also likely to be stage dependent. In early disease, it may act more as an amplifier linking abnormal innate immune sensing to B-cell-driven lesions. In later disease, it may function more as part of a maintenance network shaped by TLS maturation, redundant survival signals, and tissue remodeling (77). For this reason, it is therefore plausible that BAFF inhibition alone may fail to produce clinical benefits commensurate with biological activity in some advanced patients. In contrast, blocking IFN-BAFF coupling at an earlier stage, or intervening simultaneously with its synergistic costimulatory and B-cell survival pathways, may theoretically have a better chance of blocking this “one-way advance” pathological process (78–80).

The value of the IFN-BAFF axis, therefore, is not only that it helps explain heightened B-cell activation and autoantibody tendency in SjD, but also that it offers a framework for understanding why biological response may diverge from clinical benefit. Disease persistence may reflect not simply inflammatory intensity, but also the emergence of self-sustaining local immune circuitry. Future stratification may therefore need to integrate IFN-related features, BAFF-associated activity, TLS burden, and the degree of glandular structural preservation rather than treating all patients as the same reversible B-cell-driven disease.

## 3 The lymphoid neogenesis phase: establishment of B-cell niches and maturation of tertiary lymphoid structures

### 3.1 State-dependent sensitivity of the BAFF/APRIL axis and microenvironmental sanctuaries

In SjD, the BAFF/APRIL axis is not only involved in the regulation of B cell survival and differentiation, but also plays a central role in local immune chronicity and the maintenance of abnormal B cells. Previous studies have shown that SjD patients have elevated expression of BAFF/BLyS, and this is associated with increased disease activity, clonal expansion of B cells in salivary glands, and an increased risk of lymphoproliferative lesions, supporting a maintenance role for the BAFF-related pathway within the abnormal B-cell ecology associated with the disease (81–83). From the perspective of classical mechanisms, BAFF mainly activates the non-canonical NF-κB signaling pathway through BAFF-R, thereby supporting the maturation and survival of transitional B cells and naive B cells, which also constitute the theoretical basis for BAFF targeted therapy to have certain clinical activity in SjD (84–86).

However, accumulating evidence suggests that dependence on BAFF/APRIL signaling is not constant and may vary by B-cell state. Different B-cell subsets appear to require different survival inputs: transitional and naive B cells are more BAFF dependent early in the response, whereas more mature activated B cells and plasma cells may rely increasingly on alternative signals provided by the local microenvironment (87–90). This implies that the BAFF/APRIL axis in SjD is not simply a matter of stronger or weaker signaling, but of which B-cell subset occupies which niche and at what stage of activation.

This issue is particularly crucial in the target organs. The B cells in the salivary glands are not a homogeneous group; they are in different activated and differentiated states and are embedded in a supportive microenvironment composed of stromal cells, epithelial cells, and immune cells. Some mature B cell populations and long-lived plasma cells can receive alternative survival signals such as April, IL-6, and CXCL12 through receptors such as TACI and BCMA, thereby reducing their dependence on BAFF (91). Therefore, the persistent B cell pathology in SjD may not only reflect excessive BAFF but also indicate the formation of a tissue microenvironment that can protect mature B cells and plasma cells from exhaustion (92).

This state dependence may help explain the differences in clinical efficacy. The BELISS open-label Phase II study demonstrated that belimumab could improve the disease activity in some patients and regulate B-cell-related biomarkers, which supported the therapeutic effect of BAFF inhibition therapy in SjD (93). However, subsequent studies indicated that the use of BAFF inhibitors alone had limited effectiveness in eliminating certain mature B-cell populations (especially memory B cells); while the combination of belimumab with rituximab could more effectively eliminate B cells in peripheral blood and salivary glands (94). These results suggest that BAFF blockade may primarily affect peripheral blood and early-dependent B-cell populations, and have a smaller impact on mature B-cell populations embedded in the supportive tissue microenvironment. Animal experiments showed that the combined application of BAFF inhibition and B-cell clearance strategies could achieve stronger disease control effects, which is consistent with the above explanation (95).

From a disease-course perspective, B-cell abnormality in SjD is therefore better understood as layered rather than uniform, synchronous, or fully reversible. Early lesions may depend more heavily on BAFF-driven B-cell expansion and survival, whereas later lesions may be sustained by APRIL-rich niches, co-stimulatory signals, and TLS-associated microenvironments. This helps explain why BAFF blockade alone may be insufficient once a mature local B-cell niche has formed. Future treatment strategies may therefore need to move beyond single-pathway blockade toward more pathology-informed approaches. Combination strategies, including BAFF inhibition plus other immunomodulatory interventions such as CD40-CD40L pathway blockade, are mechanistically plausible but still require prospective clinical validation.

### 3.2 Tertiary lymphoid structures: maturity shapes function

In SjD, the formation of tertiary lymphoid structures (TLS) is not a random aggregation of inflammatory cells, but rather a programmed tissue remodeling process driven by the interaction of local chemokines, stromal cells, and immune cells. Human tissue studies support the view that TLS formation and maturation provide an anatomical context for local B-cell activation, humoral autoimmunity, and persistent gland-centered inflammation, although direct proof of causality remains limited (96, 97). In SjD, TLS maturation is closely associated with pathological humoral immunity, ectopic germinal-center-like reactions, and higher-risk tissue phenotypes.

TLS maturity appears to influence the degree of local immune organization. In the early stage, TLS often present as relatively loose aggregations of T and B cells, and the functional divisions were not yet clear. As local inflammation persists, TLS can gradually polarize and form relatively independent T cell areas and B cell areas, along with structures such as high endothelial venules (HEVs), thereby promoting the homing of circulating lymphocytes and maintaining the local immune response (98, 99). Recent studies on the spatial and single-cell landscapes of the salivary glands in SjD also suggest that the formation of TLS involves the temporal and spatial coordinated changes in the states of specific fibroblasts, perivascular cells, and immune cells, rather than a static accumulation of cells.

As TLS further mature, ectopic germinal-center-like reactions may provide a permissive setting for local autoantibody generation. In mature TLS, features related to germinal center reactions, BCL6-positive cells, actively proliferating cells, and activation-induced cytidine deaminase (AID) can be observed, suggesting that local B cells have entered the somatic hypermutation and affinity maturation process. Meanwhile, the CD21+/CD35+ follicular dendritic cell (FDC) network can form in some salivary gland lymphoid follicles, and may further support B cell activation and differentiation through antigen retention and presentation (100, 101). BCL6 is often used as a supportive marker of ectopic germinal-center-like activity in SjD salivary glands, whereas FDC network formation is generally interpreted as evidence of more mature TLS organization.

During this process, follicular helper T cells (Tfh) and peripheral helper T cells (Tph) appear to play important roles. Current studies suggest that Tfh/Tph populations can promote B-cell differentiation, plasmablast generation, and maintenance of local antibody responses through mediators such as IL-21; among them, Tph cells are of particular interest in SjD because they show high CXCL13 and IL-21 expression and are associated with local B-cell aggregation and disease activity (102–104). CXCL13 is therefore better viewed as a key mediator of B-cell chemotaxis and TLS organization that may indirectly support environments permissive for B-cell activation and plasma-cell differentiation, rather than as a direct determinant of B-cell-to-plasma-cell transition by itself.

Experimental and clinical pathological studies further support this understanding. Single-cell and spatial transcriptomic studies have revealed the structured distribution of TLS-related cell populations within the salivary glands of SjD, and have shown that there are close interactions between T cells, B cells and stromal cells. Furthermore, the maturity of TLS in salivary gland tissues, the formation of ectopic germinal centers, and the expression of related chemokines are correlated with disease activity, local high B-cell reactivity, and high-risk phenotypes. The elevation of serum CXCL13 is also associated with the histological characteristics of salivary glands, the severity of the disease, and the high-risk status of lymphoma. It may therefore be better framed as a candidate biomarker of local immune activity rather than as a validated stand-alone marker (105).

Based on the current evidence, TLS maturity is better viewed as a candidate stratification variable than as an established basis for individualized treatment in SjD. In patients with more mature TLS and stronger B-cell-driven features, targeting Tfh/Tph-B-cell interactions or CXCL13-linked tissue organization may have theoretical value, but direct clinical evidence remains limited and such approaches still require prospective validation (106).

### 3.3 Lymphoma risk: clonal evolution and microenvironmental licensing

Patients with SjD have a significantly increased risk of developing lymphoma, especially mucosa-associated lymphoid tissue (MALT) lymphoma in the parotid gland and other salivary glands. Current evidence indicates that this pathological process is not triggered by a single event, but is the result of a multi-step evolution process involving chronic self-antigen stimulation, abnormal B-cell clonal expansion, and continuous local microenvironmental support (107, 108). Under the background of continuous immune stimulation, the salivary glands may progress from reactive infiltration to B-cell clonal expansion, which is consistent with the view that “the local immune environment can provide a matrix for the subsequent occurrence of lymphoma” (109, 110).

Clonal B-cell evolution is regarded as one of the core processes underlying the occurrence of lymphomas associated with SjD. In the early stages of the disease, the infiltration of salivary glands is mainly characterized by polyclonal or multifocal B-cell proliferation; as chronic inflammation and antigen-driven persistence persist, certain B-cell clones with auto-reactive features (especially those carrying rheumatoid factor (RF) activity) can gradually acquire selective survival advantages and evolve into oligoclonal or even monoclonal expansions (111, 112). Previous studies on the immunoglobulin genes of SjD-related salivary gland MALT lymphomas have shown that these tumor cells often originate from antigen-selective auto-reactive B cells and exhibit a limited usage pattern of the heavy chain variable region gene repertoire. Although some studies have reported the preferential use of the IGHV1-69 gene, this should not be regarded as a ubiquitous molecular feature (113, 114).

The local microenvironment seems to also play a certain facilitating role in the malignant transformation process. The mature tertiary lymphoid structures and ectopic germinal centers within the salivary glands can form a microenvironment that is conducive to the continuous activation of B cells, high somatic mutation, and clonal selection. Studies have shown that the expression of activation-induced cytidine deaminase (AID), germinal center-like activity, and the formation of follicular dendritic cell networks may increase the chance for abnormal clones to acquire additional genetic events (115). At the molecular level, the dysregulation of the NF-κB pathway is considered an important factor related to the progression of lymphomas in SjD. Germline and somatic abnormalities of TNFAIP3 (A20) are associated with pSS-related lymphoma and may promote abnormal B-cell survival by weakening negative regulation of NF-κB. By contrast, t (11, 18)(q21;q21)/API2-MALT1 is better established in MALT lymphoma more broadly. Direct evidence for its role in Sjögren’s disease-related salivary gland MALT lymphoma remains limited, so it should not be overemphasized in this specific context (116, 117).

Clinical and translational studies further support this model. Longitudinal cohorts indicate that RF positivity is among the earliest and most stable predictors of lymphoma in SjD, while persistent parotid enlargement, cryoglobulinemia, hypocomplementemia, and high-risk salivary-gland histologic features further strengthen risk attribution (118). Furthermore, pathological studies have shown that SjD-related MALT lymphomas are mostly located in the salivary glands and are closely related to previous reactive lymphoepithelial lesions and clonal B-cell expansion (119).

Taken together, these findings support integrated risk assessment in SjD patients with concern for lymphoma. Histologic stratification of salivary-gland biopsies, assessment of B-cell clonality, and evaluation of germinal-center-like structures and related immunophenotypes may help identify higher-risk patients and support intensified follow-up strategies (120). At present, however, such tools are better described as useful for high-risk stratification and monitoring than as proven means of reducing lymphoma occurrence, because direct interventional evidence remains limited.

## 4 The terminal phase: stromal remodeling and functional locking

### 4.1 Structural locking: the physical barrier and rate-limiting step for functional recovery

In later-stage SjD, salivary glands may gradually develop structural changes characterized by matrix remodeling, persistent epithelial damage, and decreased regenerative capacity. These changes are associated with continued organ dysfunction (121, 122). This article uses “structural locking” as a working concept for late-stage tissue states in which local structural damage may continue to limit recovery of glandular secretion even when inflammatory activity decreases (123). Accordingly, treatment response in advanced SjD may depend not only on inflammatory activity but also on the extent of accumulated structural damage (124). Current studies further suggest disruption of epithelial homeostasis, abnormal extracellular-matrix remodeling, enhanced pro-fibrotic signaling, and impaired tissue repair, together supporting a shift from predominantly inflammation-driven injury toward tissue states in which structural damage itself becomes a major constraint (125). In particular, changes in TGF-β-related pathways, imbalance in ECM metabolism, and epithelial-mesenchymal-transition-related remodeling may exacerbate glandular sclerosis, acinar or ductal destruction, and loss of functional reserve, thereby limiting recovery with immunosuppression alone (126).

From a pathological perspective, structural locking may involve several related processes. Long-standing immune-cell infiltration and inflammatory mediator exposure can damage acinar epithelium, alter differentiation state, and impair secretion, thereby reducing both the number and functional quality of effective secretory units. Persistent inflammation may also drive extracellular-matrix remodeling and fibrosis in periductal and interstitial regions, altering tissue mechanics and potentially interfering with ductal patency and local fluid transport (126). In parallel, disruption of epithelial integrity and its supporting cellular network may further impair acinar polarity, coordinated secretion, and tissue repair. Together, these structural changes may create combined physical and biological constraints on functional recovery, helping explain why glandular improvement can remain limited despite partial inflammatory control.

Clinical observations are compatible with this view, but still require careful interpretation. Multiple studies have shown that although some biological agents can improve systemic activity indicators such as ESSDAI, the improvements in ESSPRI, dry mouth symptoms, and objective salivary secretion function are not always synchronous, suggesting that control of systemic inflammation does not always translate into recovery of glandular function (127–129). For instance, previous studies have shown inconsistency between changes in ESSDAI and ESSPRI. A review of salivary gland function also indicates that although existing biological treatments can improve local inflammation, their overall effect on restoring glandular secretion is limited (127, 130). Similarly, some novel B-cell-targeted therapies have shown benefits in terms of systemic activity or certain biological endpoints, but improvement in patient-reported symptoms and secretory function remains limited or unstable (127). In advanced disease with severe structural damage, these observations are more consistent with partial divergence between inflammation control and functional recovery than with a complete disconnection.

The imaging and pathological data further suggest that the abnormality of glandular structure itself has prognostic significance. The degree of abnormality in salivary gland ultrasound is correlated with the cumulative disease damage, decreased secretion function, and some high-risk clinical phenotypes. Therefore, the assessment of advanced SjD cannot rely solely on inflammatory activity indicators; it should also be combined with the degree of glandular structure damage, residual secretion function, and the potential window of reversibility for a comprehensive judgment (129, 131). On this basis, future treatment strategies for SjD may need to extend beyond purely anti-inflammatory approaches toward combined anti-inflammatory and tissue-repair strategies. However, this direction is currently mainly in the stage of mechanistic research and translational exploration, and lacks sufficient clinical evidence to support its routine application as a mature treatment strategy (132).

### 4.2 Fibroblast heterogeneity and epigenetic imprinting

In SjD, fibroblasts should not be viewed merely as structural support cells, but as stromal populations linked to local immune regulation, extracellular-matrix remodeling, and fibrosis progression. Single-cell transcriptome studies have revealed that fibroblasts in the salivary glands of SjD patients exhibit significant heterogeneity and are embedded in an active stromal-immune interaction network, which may jointly drive the maintenance of inflammation, epithelial regeneration impairment, and matrix imbalance (133, 134).

Functionally, these fibroblasts can be provisionally grouped into two broad states: one enriched for immune-interaction programs linked to lymphocyte recruitment, retention, and TLS organization. The other is enriched for fibrosis-execution programs, characterized by myofibroblast-like differentiation, increased extracellular-matrix production, and activation of pro-fibrotic pathways. The former can participate in the homing of local immune cells and the shaping of the microenvironment through chemokines and growth factors, while the latter is associated with the upregulation of ACTA2, POSTN and collagen-related gene expression, and may further aggravate the remodeling of glandular structure and functional limitation (135, 136).

As the disease progresses, the importance of fibroblasts that promote fibrosis may gradually increase. Current research indicates that TGF-β signaling, imbalance in extracellular matrix metabolism, and myofibroblast-like differentiation are the key driving factors in the fibrosis process of salivary glands. These changes may promote collagen deposition and tissue stiffening and may contribute to the late “structural locking” state (137).

A related issue is that sustained fibroblast activation may not depend entirely on continuing exogenous inflammatory input. Epigenetic studies in SjD suggest that DNA methylation, histone modification, and non-coding RNA dysregulation are broadly involved in disease-related gene regulation. Although direct evidence for stable stromal memory in salivary-gland fibroblasts remains limited, epigenetic mechanisms may help maintain pro-inflammatory and pro-fibrotic phenotypes once established (138, 139). It is therefore more appropriate to frame epigenetic reprogramming as a possible maintenance mechanism of persistent stromal activation than as a proven primary cause of fibrosis irreversibility.

On this basis, fibroblasts may be better understood not only as downstream consequences of structural damage, but also as plausible participants in disease persistence. Future intervention strategies may therefore consider fibroblast-state transitions, TGF-β-related pathways, and possible epigenetic maintenance mechanisms, although such approaches remain largely mechanistic or early translational in SjD and still lack sufficient clinical validation.

### 4.3 Regenerative failure and destruction of the stem cell niche

In the later stage of SjD, salivary gland dysfunction is not only associated with persistent inflammation, but also closely related to the decline in tissue regeneration capacity. Under normal circumstances, the salivary glands possess a certain capacity for renewal and repair. This process is maintained through the cooperation of duct-related stem/progenitor cells, the epithelial support network, and an appropriate local microenvironment. Current evidence suggests that in SjD this regenerative system is progressively disrupted by persistent inflammation, cellular senescence, and abnormal stromal remodeling, together contributing to reduced repair capacity (140, 141).

Inflammatory mediators are likely to be the main driving factor in this process. The continuously elevated levels of factors such as IFN-γ and TNF-α in the salivary gland microenvironment may promote immune cell infiltration, aggravate epithelial damage, activate cell death-related pathways, and weaken the tissue repair ability. Recent single-cell studies have also shown that the populations of epithelial cells and myoepithelial cells related to regeneration may undergo abnormal state transitions in SjD. For instance, the myoepithelial cell population with low expression of SOX9 is associated with impaired epithelial regeneration function, which confirms the imbalance in the regulation of the regeneration program at the disease’s advanced stage (141). Therefore, this process should be described more as the decline in the function and renewal capacity of stem cells/precursor cells rather than the depletion of the confirmed specific stem cell pool (142, 143).

In addition to inflammatory factors, the homeostasis of the stem cell microenvironment itself may also be disrupted. The extracellular matrix (ECM) of salivary gland cells not only serves as a mechanical scaffold but also participates in maintaining tissue polarity, cell adhesion, and the transmission of regeneration signals. In SjD, the abnormal remodeling of the extracellular matrix, the imbalance of basal membrane homeostasis, and the deposition of fibrotic components all change the biomechanical properties and signaling microenvironment of the local tissue, thereby weakening the microenvironmental support required for the survival and differentiation of stem/progenitor cells. Therefore, the damage to the microenvironment cannot simply be regarded as the disappearance of visible tissue structure, but should be understood as the disruption of its supporting functions.

It is worth noting that existing studies have indicated that the cell niches of salivary gland stem/progenitor cells in SjD patients show obvious characteristics of cellular senescence, and this niche senescence is associated with pathological changes in the gland and decreased secretion function. This supports the view that regenerative failure is not merely an immediate downstream consequence of inflammation, but may represent a more persistent state of repair impairment. Precisely because of this, merely controlling immune inflammation may not be sufficient to restore glandular secretion function, especially in patients with severe structural damage in the later stage, in whom the limited regenerative potential may be an important factor determining the treatment response (144, 145).

Taken together, these findings suggest that future treatment strategies for SjD may need not only to inhibit abnormal immune responses, but also to consider protection and reconstruction of the salivary-gland regenerative system, including restoration of local inflammatory balance, ECM/niche homeostasis, and residual stem/progenitor-cell repair potential. However, at present, the interventions aimed at repairing or promoting the regeneration of stem cell niches in SjD are still mainly at the stage of mechanistic research and early translational exploration. There is still a lack of sufficient clinical evidence to support their application as a routine treatment method.

## 5 Conclusion and future directions — towards stage-aware precision medicine

The simple linear inflammatory model driven solely by chronic immune activation has become increasingly difficult to explain the pathogenesis of SjD. In contrast, convergent evidence from single-cell transcriptome sequencing, spatial distribution analysis, and tissue-linked stratification studies support a more dynamic cognitive perspective: the disease process involves interactions among epithelial, innate immune, adaptive immune, stromal, and structural compartments. Under this analytical framework, the differences among different patients not only manifest in the overall severity of the disease, but also in multiple dimensions such as the dominant pathological mechanism, local immune microenvironment, tissue structure integrity, and residual functional reserve.

This review therefore proposes a stage-aware interpretive framework for SjD based on partially overlapping tissue states rather than on a single homogeneous inflammatory pathway. In early disease, epithelial innate-immune activation, viral-mimicry-like responses, and interferon-related programs may be relatively prominent. With more persistent disease, lymphoid organization, TLS maturation, and local B-cell-driven humoral activity may become more important. In later disease, matrix remodeling, fibrosis, regenerative dysfunction, and structural locking may increasingly weaken the link between inflammation control and organ recovery (146–148). The purpose of this framework is not to define a validated staging system, but to organize the above observations into working hypotheses that can be tested in future studies.

Its main translational value lies in linking mechanism to timing and endpoint selection. Precision medicine in SjD will likely require integrated stratification across clinical phenotype, serology, histopathology, imaging, and molecular readouts rather than reliance on any single indicator alone (146, 147, 149). Therapies directed at epithelial- or interferon-dominant lesions, chronic B-cell-supportive niches, and advanced structurally damaged glands are unlikely to perform similarly, and they may require different endpoint hierarchies and different expectations of reversibility.

### 5.1 Mechanistic synthesis: from linear inflammation to multi-dimensional ecological networks

Mechanistically, the framework proposed here is not a linear progressive structure. More precisely, this framework can be regarded as a dynamic network system, covering multiple aspects such as epithelial licensing, innate immune activation, lymphoid organization, B-cell-mediated humoral immune response, matrix remodeling, and regenerative dysfunction. The primary and secondary weights of these various pathological processes will change according to the location of the lesion tissue and the stage of the disease, which can also explain the inconsistency between the biological activity of drugs and clinical efficacy observed in some clinical trials of Sjögren’s syndrome. Therefore, subsequent research should prioritize the adoption of a stratified research strategy based on tissue specificity and focus on selecting gland-proximal endpoints for assessment.

### 5.2 Revolutionizing clinical trial design: mechanism-based stratification strategies

SjD is highly heterogeneous and does not fit a one-size-fits-all clinical trial model. Recent trial-design analyses and collaborative efforts, including the NECESSITY initiative, have emphasized integrating clinical, laboratory, histopathological, imaging, and biomolecular layers in order to improve recruitment, endpoint selection, and interpretation of efficacy signals (150, 151).

For recruitment, enrichment strategies should take into account both disease stage and target-organ status. Patients with relatively intact glandular structures and active interferon-related or epithelial-dominant programs may be more suitable for mechanistic immunomodulation studies. By contrast, patients with severe structural damage, fibrosis, or markedly limited functional reserve may be better suited to exploratory studies of tissue protection, anti-fibrotic intervention, or glandular regeneration. SGUS is useful in this context because it provides quantifiable indicators of structural abnormality and has been associated with secretory function, disease activity, and some lymphoma-risk features. However, its stratification role still requires prospective qualification together with careful biological interpretation (152, 153).

Endpoint selection should also be matched to the research stage, because indicators such as ESSDAI alone cannot fully reflect gland-proximal pathology, secretory reserve, or tissue-repair potential. Depending on trial purpose, the endpoint set may need to integrate target-engagement measures, organ-proximal histological or imaging parameters, glandular function, and patient-reported outcomes, with clear expectations for what can change within the selected time window (66, 150, 151, 154).

**Figure 3:**
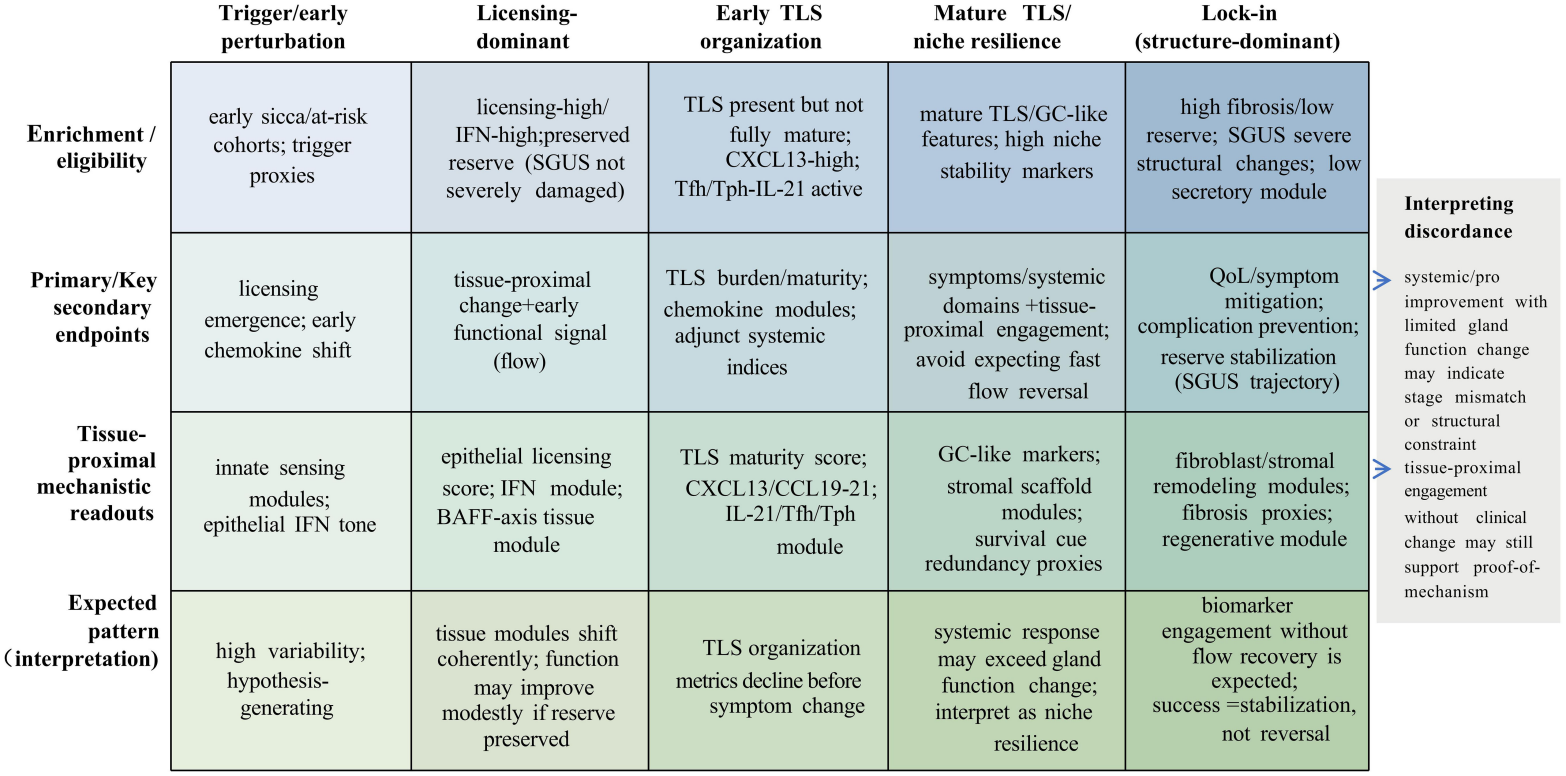
Conceptual framework for stage-matched clinical trial design in SjD. The image links putative tissue states to patient enrichment, endpoint selection, and therapeutic intent. Biomarkers such as CXCL13-related programs, epithelial antigen-presentation features, and structural imaging markers are shown as illustrative anchors. The framework is conceptual rather than a validated staging system.

Composite measures such as CRESS and STAR reflect ongoing efforts to integrate systemic activity, organ-level status, and patient-reported outcomes in SjD trial assessment (66, 154). A more informative question may therefore be not simply whether a treatment works in SjD as a whole, but in which tissue state, over what interval, and against which dominant pathological program (**Figure 3**).

On this basis, **Tables 1**–**3** should be read as illustrative, hypothesis-generating frameworks rather than as validated protocol templates. They are intended to support structured trial-design thinking, biomarker development, and interpretation of negative or mixed trial findings, but each proposed score, assay, endpoint hierarchy, and design implication would still require prospective definition and validation.

**Table 1:** Illustrative alignment of trial timescale, organ-proximal readouts, and endpoint hierarchy in stage-aware studies of SjD.

Trial timescale	Priority readouts (tissue / imaging / molecular)	Illustrative endpoint positioning	Interpretation boundaries
Short term (4–12 weeks)	epithelial licensing module score (ssGSEA), epithelial-immune interface enrichment, spatial colocalization metrics, IFN/ISG module activity, CXCL13 and BAFF levels, and exploratory SGUS measures; any short-term TLS-related signal should be treated as exploratory rather than expected	illustrative proof-of-mechanism role: target-engagement-focused endpoint positioning with exploratory enrichment for more reversible tissue states	fibrosis reversal or acinar regeneration is not expected within this timeframe; early SGUS changes may mainly reflect inflammation or edema; short-term TLS change remains speculative unless serial tissue data are available; lack of saliva-flow improvement should not be interpreted as mechanistic failure
Intermediate term (6–12 months)	TLS maturation markers (focus score, GC-like structures, BCL6, AID, Ki67), B-cell clonal expansion or Ig rearrangement signals, SGUS structural scores, fibrosis-related markers (e.g., COL1A1/COL3A1, αSMA), stromal activation programs, ductal/vascular remodeling readouts	illustrative intermediate-term role: structural or tissue-linked endpoints reflecting SGUS trajectory, TLS maturation, or inflammatory niche remodeling	disease modification may manifest primarily as slowing of progression rather than structural reversal; interpretation should consider baseline stage (reversible versus functionally locked tissue states); inflammatory and stromal readouts should ideally be interpreted together
Long term (>1 year)	functional outcomes (unstimulated/stimulated salivary flow), ocular function (Schirmer test, ocular staining score), oral disease burden (DMFT, candidiasis, recurrent parotid swelling), patient-reported outcomes, lymphoma risk surrogates (persistent parotid enlargement, monoclonal immunoglobulin, low C4, high RF)	illustrative long-term role: functional-preservation endpoints, complication tracking, and risk evaluation in enriched higher-risk cohorts	lymphoma hard endpoints usually require multi-year follow-up and high-risk enrichment; functional endpoints are strongly influenced by baseline structural reserve and should be interpreted together with intermediate structural readouts

This table provides an illustrative stage-aware framework linking trial timescale, organ-proximal readouts, and endpoint hierarchy in SjD. The listed alignments should be read as hypothesis-generating examples rather than as fixed clinical standards or validated protocol templates. Any short-term TLS-related change should be considered exploratory unless supported by serial tissue data, and feasibility differs substantially between SGUS or saliva-flow measures and biopsy-based tissue assays. Representative supporting sources include trial-design and organ-proximal studies such as JOQUER, TRACTISS, SGUS stratification work, and STAR-related outcome development (20–23, 73, 153).

**Table 2:** Operational mapping of salivary gland immune microenvironmental states to candidate readouts and endpoint positioning in SjD.

Immune microenvironmental state	Organ-proximal readouts (mechanism-priority)	Companion readouts / current feasibility (feasibility-priority)	Suggested platforms	Illustrative endpoint positioning
Triggering factors	epithelial IFN / innate immune activation; PRR / nucleic acid sensing features	blood- or saliva-based IFN and innate immune panels	bulk MSG sequencing, scRNA-seq, spatial transcriptomics, qPCR, NanoString	illustrative exploratory positioning (mechanistic validation, hypothesis generation)
Epithelial licensing	core epithelial licensing score; epithelial–immune interface enrichment; epithelial activation / antigen-presentation programs	no established low-invasive equivalent; biopsy-based multiplex epithelial IHC / ISH remains tissue-anchored and mainly exploratory	scRNA-seq, spatial transcriptomics, IF/IHC, RNAscope	illustrative exploratory-to-secondary positioning
TLS maturation	TLS maturation grading, FDC network, GC-like features, CXCL13 axis intensity, IL-21 / Tfh / Tph activity	circulating or salivary CXCL13 levels	multiplex imaging, spatial transcriptomics, ELISA, multiplex protein assays	illustrative exploratory or secondary positioning
Functionally locked decline	fibrosis burden, acinar preservation/atrophy, ductal remodeling or reprogramming	SGUS structural reserve score (OMERACT-based), unstimulated/stimulated whole saliva flow	histology with image quantification, SGUS, glandular function testing	secondary (SGUS) / possible primary for salivary-flow preservation

This table provides an illustrative operational framework linking salivary-gland immune microenvironmental states to candidate readouts and possible endpoint roles in SjD. Each candidate score or assay would still require analytic reproducibility testing, association with tissue state, incremental value beyond established markers, and explicit assessment of current feasibility before broader use. Biopsy-based multiplex IHC or ISH should be regarded as tissue-anchored exploratory assays rather than as routine clinical tools. Representative supporting evidence is drawn primarily from tissue-resolved and functional studies, including epithelial IFN/TLR work, TLS spatial analyses, CXCL13-linked tissue organization studies, stromal single-cell profiling, and SGUS stratification cohorts (27, 39, 40, 45, 63, 99, 101, 104, 106, 126, 127, 156).

**Table 3:** Lessons from representative negative or partially negative interventional studies in SjD.

Trial or program	Biological signal observed	Why the primary clinical endpoint may have failed	Illustrative implication for trial design
Hydroxychloroquine (JOQUER)	Downregulation of systemic interferon-related activity with limited effect on objective glandular outcomes	Symptom-centered primary endpoint, heterogeneous enrollment, and weak linkage between systemic modulation and local tissue state	Illustrates that systemic anti-inflammatory activity should not be assumed to predict glandular recovery
Rituximab (TRACTISS and related RCTs)	B-cell depletion with some biomarker and ultrasound changes	Mature tissue damage, incomplete disruption of tissue niches, and endpoints misaligned with the likely repair window	Illustrates the value of enriching for more reversible tissue states and pairing B-cell depletion with organ-proximal endpoints
Belimumab or BAFF inhibition	Biomarker changes and biological activity signals in open-label or sequential studies; inconsistent symptomatic benefit in randomized settings	Pathway redundancy, persistence of niche-embedded mature B cells, and intervention that may occur after chronic niches are established	Illustrates why tissue-linked eligibility criteria and rational combination or sequential strategies may merit testing
Co-stimulation blockade (abatacept- or anti-CD40-type programs)	Immune modulation in selected subsets	Blockade of T-cell-help pathways may be insufficient once TLS maturation and stromal damage are established; partial biological effects may not be captured by global endpoints	Illustrates the importance of matching pathway choice to the dominant microenvironmental program and pre-specifying mechanistic secondary endpoints

This table provides an illustrative interpretive summary of representative negative or partially negative interventional studies in SjD. It is intended to highlight possible reasons for trial failure, including tissue-stage mismatch, pathway redundancy, endpoint misalignment, and limited structural reversibility, rather than to rank efficacy across studies. Key supporting interventional references include JOQUER, randomized rituximab studies, TRACTISS and its SGUS substudy, BELISS, the iscalimab proof-of-concept trial, abatacept phase III data, and sequential belimumab-rituximab studies (20–23, 62, 73, 78, 80, 94, 141, 153, 154).

### 5.3 The quest for novel biomarkers: from serology to histology

To implement precision medicine in SjD, there is a need for biomarker frameworks that can support patient stratification, response monitoring, and context-specific interpretation. Traditional serological markers such as anti-Ro/SSA and anti-La/SSB remain diagnostically valuable, but they do not precisely distinguish disease stage, local tissue state, or treatment response. Their interpretation therefore needs to be integrated with imaging, histopathology, and molecular readouts (146, 147, 149). Among complementary tools, SGUS is currently one of the most scalable candidates. Available studies show that salivary gland ultrasound reflects structural abnormality and is associated with secretory function, disease activity, and some lymphoma-risk features. With OMERACT-based scoring and multicenter standardization, its value for stratification and longitudinal follow-up has improved. Even so, careful biological interpretation remains necessary (153, 154).

The biopsy of minor salivary glands still holds significant reference value when it comes to studying the state of epithelial cells, the maturity of tertiary lymphoid structures, the local arrangement of B cells, the remodeling of the matrix or the assessment of functional reserves related to fibrosis (155). Based on the histological and molecular data sets obtained from the biopsy, it can provide support for patient stratification in clinical trials, mechanism verification, and the development of biomarkers. When conditions permit, flow cytometry or high-dimensional omics techniques can also be used to conduct tests on the minor salivary gland tissues. However, at present, these methods are more suitable for mechanism exploration and stratification studies, and are not yet suitable for routine clinical assessment.

Therefore, the goal is not to search for a single universal biomarker, but to build integrated detection panels matched to specific research or clinical purposes. From this perspective, the role of **Table 2** is not simply to list candidate readouts, but to provide an illustrative template linking tissue-state hypotheses, assay platforms, and endpoint positioning.

### 5.4 Final remarks

Stage-aware and mechanism-based stratification remains promising, but its value will depend on prospective validation, explicit operationalization of candidate scores, and more informative organ-proximal endpoint strategies. The practical goal is to match the right intervention to the right patient within a biologically plausible disease window and to evaluate it with mechanism-relevant endpoints.
